# HPLC-MS/MS Method for the Detection of Selected Toxic Metabolites Produced by *Penicillium* spp. in Nuts

**DOI:** 10.3390/toxins12050307

**Published:** 2020-05-08

**Authors:** Davide Spadaro, Giovanna Roberta Meloni, Ilenia Siciliano, Simona Prencipe, Maria Lodovica Gullino

**Affiliations:** 1 Centre of Competence for the Innovation in the Agro-Environmental Sector (AGROINNOVA), University of Torino, Largo P. Braccini 2, 10095 Grugliasco (TO), Italy; giovannaroberta.meloni@unito.it (G.R.M.); ilenia.siciliano@unito.it (I.S.); simona.prencipe@unito.it (S.P.); marialodovica.gullino@unito.it (M.L.G.); 2 Department of Agricultural, Forestry and Food Sciences (DISAFA), University of Torino, Largo P. Braccini 2, 10095 Grugliasco (TO), Italy

**Keywords:** almond, chestnut, hazelnut, mycotoxins, *Penicillium* spp., walnut

## Abstract

*Penicillium* spp. are emerging as producers of mycotoxins and other toxic metabolites in nuts. A HPLC-MS/MS method was developed to detect 19 metabolites produced by *Penicillium* spp. on chestnuts, hazelnuts, walnuts and almonds. Two extraction methods were developed, one for chestnuts and one for the other three nuts. The recovery, LOD, LOQ and matrix effect were determined for each analyte and matrix. Correlation coefficients were always >99.99%. In walnuts, a strong signal suppression was observed for most analytes and patulin could not be detected. Six strains: *Penicillium bialowiezense*, *P. brevicompactum*, *P. crustosum*, *P. expansum*, *P. glabrum* and *P. solitum*, isolated from chestnuts, were inoculated on four nuts. Chestnuts favored the production of the largest number of *Penicillium* toxic metabolites. The method was used for the analysis of 41 commercial samples: 71% showed to be contaminated by *Penicillium*-toxins. Cyclopenin and cyclopenol were the most frequently detected metabolites, with an incidence of 32% and 68%, respectively. Due to the risk of contamination of nuts with *Penicillium*-toxins, future studies and legislation should consider a larger number of mycotoxins.

## 1. Introduction

Nuts are important components of the Mediterranean diet and their consumption is widespread all over the world. In 2018, Italy was the second largest European chestnut producer with 53,280 t/year, and the second largest world producer of hazelnut with 132,699 t/year. The production of almonds, walnuts and pistachios was 79,801 tons, 12,173 tons and 3864 tons, respectively [1].

Nuts are susceptible to molds because of their characteristics of water activity, moisture, nutrient content, pH and long storability. The most common fungi detected on nuts are *Aspergillus* spp., *Rhizopus* spp., *Penicillium* spp. [2,3]. Potentially toxigenic species, such as *Aspergillus flavus*, *A. parasiticus,* and *A. niger*, have been often reported on nuts [4,5]. These species are well known as producers of aflatoxins and ochratoxins, with genotoxic and carcinogenic properties [6,7]. Due to their proved toxicity, the European Commission set maximum levels for aflatoxins in nuts (Commission Regulation (EU) No. 165/2010). No legislation is in force in Europe to regulate the presence of other important mycotoxins on nuts produced by other fungal species. Species of *Penicillium*, for example, are well known to be contaminant of different foodstuff, including nuts. Overy et al. [8] and Prencipe et al. [9] reported the presence of *P. crustosum*, *P. glabrum* and *P. discolor* on fresh chestnuts, linked to the production of penitrem A (PenA), ochratoxin A (OTA) and chaetoglobosin A (ChA). Furthermore, Prencipe et al. [9] reported the production in vivo of different secondary metabolites by species of *Penicillium* isolated from chestnuts: cyclopenin (CPN), cyclopenol (CPL), viridicatin (VIR) and viridicatol from *P. crustosum*, *P. discolor*, *P. polonicum* and *P. solitum*; roquefortine C (RoqC), patulin (PAT), andrastin A (AndA) and mycophenolic acid (MPA) by *P. crustosum*, *P. expansum*, *P. gladicola* and *P. bialowiezense*, respectively. Production of PenA by *P. crustosum* was also described by Richard et al. [10] on walnuts. The presence of *P. chrysogenum* and *P. citrinum* was reported on hazelnuts and walnuts by Abdel-Hafez and Saber [11]. *P. brevicompactum*, *P. crustosum*, *P. glabrum* and *P. solitum* were reported on walnuts by Tournas et al. [12]. PAT, RoqC and ChA produced by *P. expansum* were reported on nuts in 2004 [13]. These are just a few examples of *Penicillium* spp. contamination reported in the literature, highlighting the importance of further studies about the presence of toxic metabolites on nuts. Other toxic secondary metabolites produced by *Penicillium* spp. on nuts, include citrinin (CIT), cyclopiazonic acid (CPA), citreoviridin (CVD), meleagrin (MEL), asterric acid (AsA), sulochrin (SUL), penicillin V (PenV), penicillin G (PenG) and griseofulvin (GRI).

Among these compounds, most are known for their toxic effects, such as cytotoxicity, mutagenicity, carcinogenicity, teratogenicity, nephrotoxicity, neurotoxicity, tremorgenicity and immunosuppression. Some metabolites have also pharmaceutical and anti-tumor activities [14]. OTA is a nephrotoxin and is believed to cause urinary tract tumors and Balkan endemic nephropathy in humans [15,16]. Indeed, levels of OTA must be as low as possible to avoid its potential toxic effects, including teratogenicity [17] and carcinogenicity [18]. Exposure to PAT is associated with immunological, neurological and gastrointestinal outcomes [19]. Although used in many medical and veterinary products for antifungal and anti-proliferative activity [20,21], GRI has been classified as a potential carcinogenic for humans by the International Agency on Research on Cancer [22].

The mycotoxigenic potential of fungi depends on species and strains, matrix composition and environmental factors, such as temperature and moisture [23]. However, even on identical substrates, the complex interaction of several factors may provide significant variations in fungal growth and mycotoxin production [24]. The presence of various species belonging to *Penicillium* spp. is of great concern because of their capability to produce a large number of mycotoxins and other noxious metabolites [12].

Given the possible risks for public health and the increase of restrictive regulations for mycotoxin in food, the development and validation of robust methods for the determination of mycotoxins are increasingly useful. Several methods for the screening or quantitative analyses of a large number of metabolites have been developed and validated for different matrices and methods, based on multi-mycotoxin detection, and are replacing methods for single analytes [25,26,27]. Most of these methods are based on liquid chromatography systems (HPLC or UHPLC) equipped with tandem mass spectrometry. Sulyok and colleagues [27] published the extension of an existing LC-MS/MS multi-mycotoxin method, which includes the detection of 87 analytes used for a semi-quantitative screening of bread, fruit, vegetables, cheeses, jam and nuts. Varga et al. [28] developed and validated a semi-quantitative UHPLC-MS/MS method for the determination of 191 mycotoxins and other fungal metabolites in pistachios, almonds, peanuts and hazelnuts.

In this study, the aim was to develop and validate a HPLC-MS/MS method for the simultaneous determination and quantification of 19 metabolites produced by *Penicillium* spp. on hazelnuts, almonds, chestnuts and walnuts. The selected metabolites were 10 mycotoxins (CIT, CPA, CVD, GRI, MEL, MPA, OTA, PAT, PenA and RoqC), 3 antibiotics (PenG, PenV and SUL), and other toxic secondary metabolites (AndA, AsA, ChA, CPN, CPL and VIR). The method performance parameters, such as matrix effects and recoveries, were investigated. Once optimized and validated, six different *Penicillium* spp. isolated from chestnuts were selected (*P. brevicompactum*, *P. bialowiezense*, *P. crustosum*, *P. expansum*, *P. solitum* and *P. glabrum*) for artificial inoculation of four nuts (chestnuts, hazelnuts, walnuts and almonds) to test the method. Finally, the analytical method was tested on commercial nut samples, for the possible detection of the selected secondary metabolites.

## 2. Results and Discussion

At first, fragmentation of selected *Penicillium*-toxins was investigated using pure standards, and mass spectrometric parameters were optimized for all analytes in both positive and negative ESI mode, and then, the best polarity was chosen for each compound. Each metabolite, when fragmented, shows a characteristic mass spectrum with a precursor ion and product ions. Two specific product ions were selected for each target compound and the most abundant one was used for quantitation while the second product ion was used for confirmation. To optimize the tuning experiments, the ion selection as well as the ion transport through the mass spectrometer are performed by direct infusion of a standard solution at 1 μg/mL of each analyte with a syringe pump at a flow rate of 20 μL/min. Analyst software-supported protocols were sufficient for tuning the instrument.

Polarity, precursor, and product ions and collision (eV) voltages for mass spectrometric analyses are presented in Table 1.

Chromatographic conditions were optimized by using different eluents and several gradients to obtain the best separation for each analyte. The chromatogram was segmented into four different time sections, and only a limited number of MRM transitions were scanned within these periods to increase the repeatability of the analysis. To evaluate the linearity, a seven-point calibration curve was constructed for each analyte in the blank matrices. The calibration curves constructed throughout the study were linear. All the calculated correlation coefficient (R^2^) values were > 99.99%. This confirmed the linearity of the analytical range [29] (Table 2, Table 3, Table 4 and Table 5).

The specificity and selectivity were examined using blank and spiked extracts of each analyzed matrix. Twenty-five injections per blank matrix were made to monitor the interference peaks in the ion chromatograms, where there is no significant interference at the retention times of each analyte.

Sample preparation is of primary importance in mycotoxin determination because it affects every chromatographic determination. The most common solvents used for the extraction of mycotoxins from foodstuff are methanol-water and acetonitrile-water [26,28,30]. In addition, other solvents, such as acetone [31,32], ethyl acetate [33,34] and chloroform [35,36], are also used for mycotoxin extraction. The conditions for the extraction were subjected to an optimization process, which involved the selection of the type of extraction solvent (ethyl acetate, chloroform, methanol, acetonitrile, toluene, two different mixtures of water:methanol, and a mixture of water:acetonitrile) and the volume used (15 and 30 mL); solvents were used in single or in multiple steps in order to identify the best extraction mixture (data not shown).

Different acids were added to modify the pH (HCl 37% and HCOOH 0.1%, CH3COOH 0.1%, or none), methods of stirring (ultrasonic bath or rotary shaker) and incubation times (15, 30, 45, 60 and 90 min) were also tested (data not shown). Extraction conditions can be greatly influenced by matrix composition and a unique method is not always suitable for all types of food matrices. Since ESI ionization may be subject to signal suppression or enhancement due to coextracted matrix constituents, the validation study included the evaluation of matrix effects.

The peak heights and areas determined for the analytes were found to be dependent on the matrix. A signal enhancement was observed for CPN, CPL, PenA, RoqC, GRI and AsA in hazelnuts; for CPN, CPL and PenA in chestnuts; and only for PenA in almonds. In walnuts, a strong signal suppression was observed for most analytes and PAT could not be detected. These results suggested that matrix-matched calibration is necessary for the accurate quantification of metabolites in these matrices. As the matrices used in this work have different chemical compositions, it was not possible to use a unique extraction method. The best extraction solvent for each matrix was chosen by comparing recovery data for every extraction mixture used; indeed, during sample preparation, two different extraction methods were developed to improve the recovery of each analyte, taking into account different matrix composition and chemical structures of the metabolites. In particular, chestnuts were compositionally different compared to the other nuts investigated. Chestnut carbohydrates quantities ranged from 75% to 86%, while total fat content ranged from 0.5% to 2% [37] and water represented approximately 50% of the content [38]. Almond kernel has an extremely low water content (4%–6%) and high levels of proteins (18%), fats (54%) and carbohydrates (20%) [39]. The main constituents of walnuts are fats ranging from 79% to 82%, dietary fiber ranges from 4% to 5%, while starch content is lower than 2.8% [40]. Hazelnut oil is rich in vitamin E and fatty acids, in particular oleic acid. Among the fatty acids of hazelnut oil, palmitic acid (2.96–7.40%) is the main saturated fatty acid (SFA). The highest share of fatty acids is monounsaturated fatty acids (MUFAs), as oleic acid (73.1%–90.7%), and polyunsaturated fatty acids (PUFAs) as linoleic acid (4.4–16%) [41,42]. Due to the uniqueness of the matrices, the composition of the solvent applied for extraction is a crucial parameter during the development of a multi-mycotoxin method.

Different mixtures of organic solvents and water, sometimes with the addition of modifiers, such as acids or bases, are commonly used for the extraction of mycotoxins, depending on their physicochemical properties [43]. The accuracy was measured based on the recovery of standard compounds in matrices. The average recoveries were evaluated by calculating the ratio of the amount detected versus the amount injected. According to Commission Regulation (EC) No 401/2006 of 23 February 2006 [44], typically a recovery within the range 70–110% is required. The mean recoveries for compounds were from 69.4% (CPA) to 93.4% (AndA) for chestnuts, 64.6% (RoqC) to 104.2% (GRI) for hazelnuts, 68.2% (AndA) to 98.1% (CPA) for walnuts and 66.3% (PenA) to 96.2% (CVD) for almonds. The addition of a strong acid (HCl) was tested, and, in this condition, CPL, CPN, ChA, and PenA were not detected (data not shown), while the use of 0.1% of formic acid induced an increase in the extraction performance.

The results concerning LOD and LOQ depended on the analytes and matrices. CPL showed always the best detection and quantification limits, while PenG had the highest limits of quantification on chestnuts, almonds and walnuts. On hazelnuts, ChA showed the highest LOD and LOQ.

In order to evaluate the precision, intra-day and inter-day tests were assessed on a single day and on three consecutive days, respectively. Table 6 shows how all coefficient variability values were ≤ 15%, which were in compliance with the legislation [44], suggesting that the method is robust.

The method developed was applied to artificially and naturally contaminated nuts. In order to investigate the co-occurrence of different metabolites and if the quantitative determination of analytes on real samples is possible, six different *Penicillium* spp., isolated from chestnuts, were selected based on their metabolic profile [45] and inoculated on the matrices: *P. bialowiezense*, *P. brevicompactum*, *P. crustosum*, *P. expansum*, *P. glabrum* and *P. solitum*.

**Figure 1:**
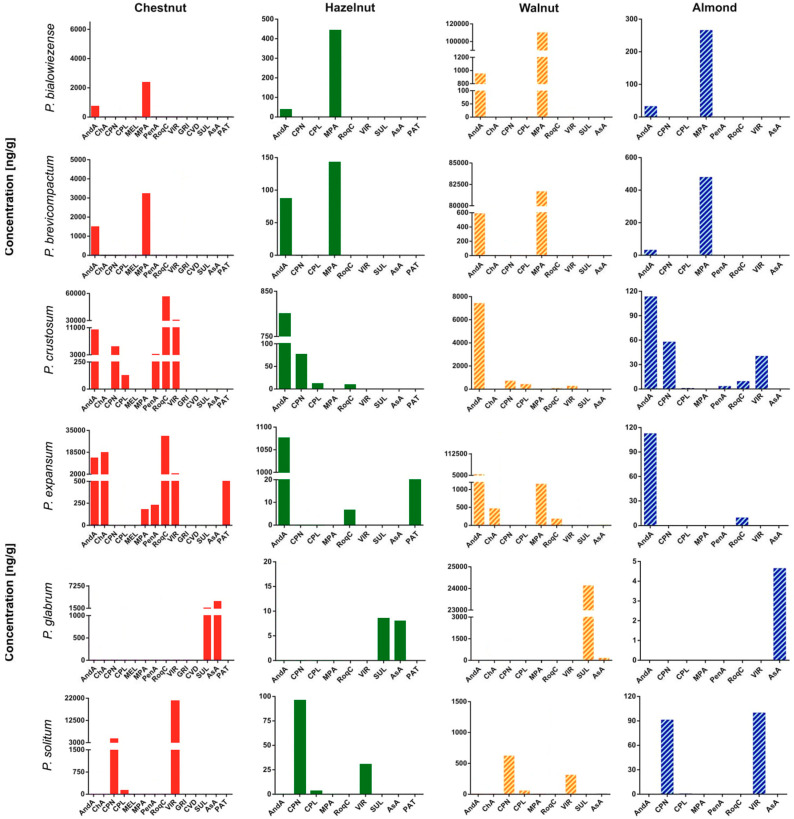
Production of secondary metabolites by *P. bialowiezense, P. brevicompactum, P. crustosum, P. expansum, P. glabrum* and *P. solitum* on chestnuts, hazelnuts, walnuts and almonds.

*P. bialowiezense* and *P. brevicompactum* are closely related and are morphologically, genetically, and chemically similar; they are known for their ability to produce MPA and derivatives, asperphenamates, AndA, quinolactacin A, citreohybridonol, and Raistrick phenols [46]. Among the metabolites investigated, MPA and AndA were produced on the four matrices by *P. bialowiezense* and *P. brevicompactum* (Figure 1).

On hazelnuts and almonds, the production of MPA and AndA by *P. bialowiezense* was similar, and these were the only produced metabolites [8,47,48]. On walnuts, the production of MPA was more abundant, and AndA was also produced. *P. brevicompactum* showed on hazelnuts, walnuts and almonds a similar behavior to *P. bialowiezense*.

*P. crustosum* is reported to produce RoqC, cyclopeptin, cyclopenins, viridicatins, penitrems, AndA, terrestric acid, hadacidin, 2-methyl-isoborneol and palitantin [49,50]. In our study, RoqC, cyclopenins, viridicatins, penitrems, AndA on chestnuts were detected. As reported by Prencipe et al. [9], several species able to produce these mycotoxins were isolated from chestnuts with a high incidence. On the other matrices, AndA was the main metabolite with a production of 804 ng/g on hazelnuts, 7390 ng/g on walnuts and 113 ng/g on almonds (Figure 1).

AndA and RoqC were produced on the four matrices, PAT was detected only on chestnuts and hazelnuts. ChA was produced only on chestnuts (18,600 ng/g) and walnuts (470 ng/g). CIT and OTA were not detected in any of the inoculated matrices. Our results on inoculated matrices with *P. expansum* are consistent with those already reported in the literature [51,52].

AsA was produced on the four tested matrices inoculated with *P. glabrum* and SUL was produced on chestnuts, hazelnuts and walnuts, according to Barreto et al. [53] and Prencipe et al. [9] regarding fresh chestnuts and derivatives.

Cyclopenins and VIR were produced on all the matrices inoculated with *P. solitum*, a species known to produce these secondary metabolites [45].

None of the selected species were able to produce CVD, penicillins, MEL and CPA, which could be produced by other species of *Penicillium* spp. reported on nuts [45]. As reported by Prencipe et al. [9] the occurrence of many *Penicillium* species is found in fresh chestnuts, which are able of producing various metabolites, while Frisvad et al. [48] reported the frequent presence of *P. discolor*.

The production of metabolites depends on the matrix. In fact, on chestnuts, 11 analytes were detected, while on almonds, 8 analytes were detected at concentration lower than on chestnuts. Chestnuts favored the production of more *Penicillium*-toxins. This could be explained by the origin of the strains; all of them are isolated from chestnuts. In addition, the composition of chestnuts, rich in carbohydrates, could be another factor favoring the growth of *Penicillium* spp.

In order to validate the analytical method, 41 commercial samples were analyzed for simultaneous determination of the 19 *Penicillium* metabolites. Table 7 shows the detected metabolites (above LOD) in the analyzed samples, the incidence of positive samples, the minimum and the maximum level of each analyte in different matrices. Nine of the 19 metabolites investigated were detected in commercial nut samples and 29 out of 41 samples (70.7%) were positive for at least one of the analytes considered. CPN and CPL were the most commonly found analytes in all matrices, and CPN levels were always lower than CPL. The highest levels of CPN and CPL were observed in chestnut samples with a concentration of 105.7 and 1106.01 ng/g, respectively. CPN and CPL are produced by different *Penicillium* species such as *P. crustosum*, *P. discolor* and *P. solitum*, which have generally been reported as important contaminants in almonds, chestnuts, walnuts and hazelnuts [9,40,47,48,54]. Furthermore, CPN serves as a precursor for CPL, which explains the frequent co-occurrence of these extrolites in the matrices considered. CPN and CPL, as well as VIR, the latter detected in two samples out of 41 (almonds and walnuts), belong to the class of benzodiazepine alkaloids [55], and they have ecological significance due to their phytotoxic and antimicrobial properties [56,57]. Varga and colleagues [28] found VIR contamination in nut samples, with an incidence of 43% and a high contamination levels of MPA in hazelnuts and low contamination levels of CPA in almonds, recording an incidence of 47% and 13%, respectively. In the commercial nuts analyzed, MPA was detected in three matrices, but not in chestnuts. Although MPA has several bioactivities (antibiotic, antifungal, antiviral, and antitumor) [58], its consumption may affect the human immune response, causing a higher incidence of bacterial infections [59].

RoqC was present in all matrices, with a high incidence (62.5%) in chestnuts, however, the highest level was found in walnuts (118.95 ng/g), while PenA was found only in chestnuts. Several studies reported RoqC and PenA contaminations in nuts. Frisvad and Samson [47] reported PenA and RoqC from *P. crustosum* growing on cheese or nuts, Prencipe et al. [9] showed RoqC and PenA production on inoculated chestnuts by different *Penicillium* species and Bertuzzi et al. [60] reported the occurrence of RoqC in fresh chestnuts. RoqC was also found in serum and urine samples collected from a dog that had a history of ingesting moldy walnuts [61].

ChA was frequently found in chestnut samples, recording a concentration range between 6.45 and 376.35 ng/g, compared to almonds and hazelnuts, where a low concentration of this contaminant was registered (Table 7). Indeed, ChA, B and C could potentially be produced from *P. discolor* in several foodstuffs and have been found as natural contaminants in chestnuts [8].

AndA produced by *P. crustosum* [47] was detected in chestnuts and walnuts, while SUL produced by *P. glabrum* [53] was found only in chestnut samples. For both metabolites, their production was recorded in nuts [9].

In conclusion, chestnut samples showed natural contamination with seven target analytes detected (AndA, ChA, CPN, CPL, PeA, RoqC and SUL), a number higher than walnut, hazelnut and almond samples. For walnut samples, six analytes (AndA, CPN, CPL, MPA, RoqC, and VIR) were detected, while five for hazelnut and almond samples (CPN, CPL, MPA, RoqC and VIR and ChA, CPN, CPL, MPA and RoqC, respectively).

The analysis of commercial samples suggests that chestnuts favored the production of the largest number of *Penicillium* metabolites, confirming that probably its composition, richer in carbohydrates, seems an important factor for the growth of *Penicillium* spp. and consequent mycotoxin production.

The most frequently detected metabolites were CPN and CPL, present in over 32% and 68% of the samples. CPL was found in all matrices, suggesting that it can be considered as an indicator of the *Penicillium* spp. contamination in nuts.

## 3. Conclusions

The methodology reported in this study enables the determination of different *Penicillium* metabolites in four nut matrices. The selectivity and sensitivity of the LC-MS/MS method allow for the simultaneous investigation of different metabolites, often present at low concentration. The application of this method to commercial samples could help to evaluate the risks associated with the presence of toxic metabolites produced by *Penicillium* spp.

Furthermore, this method could be helpful in the identification of species of *Penicillium* based on their metabolic profile [62]. The metabolic profile of *Penicillium* spp. has been often used to characterize and identify the different species of the genus [63], but differences could occur from isolate to isolate [64]; therefore, the application of a quantitative method could be useful to understand the uniqueness of each strain, but also its closeness to other isolates. 

Not all detected metabolites produced by *Penicillium* spp. are toxicologically relevant and create health concerns to consumers at their naturally occurring levels [65]. However, toxicological tests tend to consider the effects of a single metabolite. As fungi can produce several toxic compounds at the same time [66], future toxicological studies should consider the toxic effects of several fungal metabolites together and this method could help to monitor and study mycotoxins and other metabolites produced by *Penicillium* spp. in nuts. Due to the possible fungal contaminations on nuts, a revision of the current legislation should include a larger number of *Penicillium* toxins to prevent the risks for consumers associated to nut contaminations.

## 4. Materials and Methods

### 4.1. Matrices and Chemicals

Chestnuts, hazelnuts, walnuts and almonds were purchased in an Italian store, and analyzed to evaluate the absence of the analytes (Supplementary Table S1). Analytical standards for viridicatin (≥98%), citrinin (≥98%), cyclopiazonic acid (≥98%), citreoviridin (≥97%), penitrem A (≥97%), andrastin A (≥95%), ochratoxin A (≥98%), patulin (≥98%), meleagrin (≥98%), mycophenolic acid (≥98%), asterric acid (≥98%) and sulochrin (≥99%) were obtained from Enzo Life Sciences (Rome, Italy). Analytical standard for roquefortine C (>99%) was obtained from Bioaustralis (Smithfield, NSW, Australia). Analytical standards for penicillin V (≥98%), penicillin G (≥98%), chaetoglobosin A (≥98%), griseofulvin (≥97%), cyclopenol (≥95%) and cyclopenin (≥95%) were obtained from Sigma-Aldrich (St Louis, MO, USA). Stock solutions were prepared for each analyte at the concentration of 1 mg/mL and the working solution was prepared by dilution up to 2 µg/mL by mixing the stock solutions of each analyte. Stock and working solutions were stored at −20 °C until use, while calibration solutions were prepared daily by using the working solution mixture and diluting with acetonitrile:water (50:50, v/v) or blank matrix. Water was obtained from a Milli-Q system (G. Maina, Pecetto Torinese, Italy). Methanol, acetonitrile (VWR International, Radnor PA, USA), ethyl acetate, dichloromethane and toluene (Sigma-Aldrich) were HPLC-grade. Formic acid, acetic acid and hydrochloric acid (Sigma-Aldrich) were analytical reagent-grade.

### 4.2. Metabolite Extraction Protocols

#### 4.2.1. Extraction from Chestnuts

Ten grams of homogenized fresh chestnuts were extracted with 20 mL of acetonitrile acidified with 0.1% formic acid on a horizontal shaker (700 rpm) for 30 min. Liquid was collected in a centrifuge tube. Samples were then extracted with 20 mL of ethyl acetate on a horizontal shaker (700 rpm) for 30 min. Liquids were combined in centrifuge tubes. The extract was evaporated to dryness and reconstituted to a final volume of 500 µL with a mixture of acetonitrile:water (50:50) (v/v).

#### 4.2.2. Extraction from Hazelnuts, Walnuts and Almonds

Ten grams of each nut species were used for the extraction with 15 mL of methanol acidified with 0.1% formic acid and shaken at 700 rpm on a horizontal shaker for 30 min. Liquid was transferred to a new centrifuge tube. Solid samples were then extracted with 15 mL of acetonitrile acidified with 0.1% formic acid on horizontal shaker (700 rpm) for 30 min. Liquid was collected and added to the first extract. Solid samples were finally extracted with 15 mL ethyl acetate for 30 min at 700 rpm on a horizontal shaker. Liquid extracts were mixed together and evaporated to dryness and reconstituted to 500 µL with a mixture of acetonitrile:water (50:50) (v/v).

### 4.3. HPLC-MS/MS Analysis of Metabolites

Analyses were performed using a 1260 Agilent Technologies system consisting of a binary pump and a vacuum degasser, connected to a Varian autosampler, Model 410 Prostar (Hansen Way, CA, USA), equipped with a 20 μL loop coupled to a Varian 310-MS TQ Mass Spectrometer. The separation of mycotoxins was performed using a Gemini-NX C18 (150 × 2 mm, 3 μm, Phenomenex, Torrance, CA, USA) under a flow of 200 μL/min. Solvent A was H_2_O acidified with formic acid 0.05%, while solvent B was CH_3_CN. Elution gradient started with 30% of solvent B for 5 min, increased to 50% in 10 min and remained at 50% for 10 min, then increased to 100% in 20 min. During the next 6 min, the column was washed and readjusted to the initial conditions and equilibrated for 10 min. The volume of the injection was 10 μL. Samples were ionized using an electrospray (ESI) ion source operating in positive and negative ion modes in different segments. For the multiple reaction monitoring (MRM) experiments, two transitions were selected for each compound (Table 1) and the collision gas (Ar) pressure was set at 2 mbar for all the experiments.

### 4.4. Validation of Analytical Parameters

The method was validated according to EN ISO/IEC 17025:2017 [29] and performance criteria reported in Commission Regulation (EC) 401/2006 [44], for the 19 toxic metabolites produced by *Penicillium* spp., using the linearity range of parameters, limits of quantification (LOQ) and detection (LOD), recovery, matrix effect (ME), specificity and selectivity.

#### 4.4.1. Linearity Range

The analytical curves were constructed for each substance from different dilutions in the mobile phase. Seven concentration points were included in the range of 10–200 ng/mL for all the analytes. Analytical curves were also built by using the matrix extracts from chestnuts, hazelnuts, walnuts and almonds.

#### 4.4.2. Limit of Detection and Limit of Quantification

LOD and LOQ were estimated from the linearity of the calibration curves. They were determined based on the slope and standard deviation (σ) of the linear coefficient of the analytical curve: LOD = 3 × (σ × linear coefficient)/slope and LOQ = 10 × (σ × linear coefficient)/slope.

#### 4.4.3. Recovery

Recovery experiments consisted of spiking the blank matrix with the standards. To obtain the percent recovery, the equation Recovery = [observed concentration of spiking sample]/[expected concentration] × 100 was used.

#### 4.4.4. Matrix Effect

To determine the matrix effect, analyses were performed using the standards in matrix and in solvent. The extract used for this trial was previously analyzed, and the analytes of interest were absent. To check for a matrix effect, the extract was spiked with the standards at seven concentrations, the same used for linearity range analysis (from 10 to 200 ng/mL). The proportion of the matrix effect (ME) was calculated from the equation: ME = [slope (analyte in extract)/slope (analyte in solvent)] × 100.

#### 4.4.5. Specificity and Selectivity

Selectivity was assessed by comparing the chromatograms obtained from the injection of blank and spiked extracts with the analytes of interest. Occurrence of coeluting interferences arising from the matrix with the analyte of interest was evaluated.

#### 4.4.6. Precision

Precision was evaluated performing intra-day and inter-day tests. Intra-day tests were carried out by fortifying blank matrix at 50 ng/g of each analyte and injecting ten times on a single day. For inter-day tests, this protocol was repeated on three consecutive days. The precision was evaluated by coefficient variability values (CV%).

### 4.5. Artificial Contamination of Matrices

Six *Penicillium* spp. strains (Supplementary Table S2) isolated from chestnuts, identified by molecular and morphological analyses and capable of producing some of the analytes investigated [9], were selected for artificial contamination of the matrices. The selected strains belonged to *P. bialowiezense*, *P. brevicompactum*, *P. crustosum*, *P. expansum*, *P. glabrum* and *P. solitum*. Fungal spore suspensions were prepared by adding Tween suspension (1%) to fungal plates cultured for 7 days on Potato Dextrose Agar. Nut samples were surface disinfected by immersion in 1% hypochlorite solution, washed with distilled water and air-dried. Then, they were transferred into petri dishes, and six plates were used for each type of nut. Five holes (3 mm depth) were made on each nut, and each hole was inoculated with 5 µL of *Penicillium* sp. Suspension (1 × 10^5^ CFU/mL). The plates were incubated at 25 °C for 1 week.

### 4.6. Commercial Samples Analysis

To check the applicability of the developed method, 41 nut samples (Supplementary Table S1), collected randomly from local markets in Turin (Italy), were sampled for mycotoxins analysis. Samples purchased included eight lots of chestnuts, 11 of almonds, 13 of hazelnuts and nine of walnuts. The quantification of the commercial nut samples was performed against the corresponding matrix-matched calibration curves approved with acceptable R^2^.

## Figures and Tables

**Table 1:** Polarity, precursor and product ions with collision energy (eV) and retention times for the analytes used for mass spectrometric analyses. Product ions used as quantifiers were reported in bold.

Compound	Abbreviation	Formula	Retention Time (min)	Precursor Ion	Product Ion (Collision Energy, eV)
Andrastin A	AndA	C_28_H_38_O_7_	34.61	487 (+)	243(22)/427(12)
Asterric acid	AsA	C_17_H_16_O_8_	22.02	347 (−)	149(12)/256(16)
Chaetoglobosin A	ChA	C_32_H_36_N_2_O_5_	29.25	529 (+)	130(28)/511(12)
Citreoviridin	CVD	C_23_H_30_O_6_	21.94	403 (+)	139(24)/297(16)
Citrinin	CIT	C_13_H_14_O_5_	20.81	251 (+)	233(16)/205(20)
Cyclopenin	CPN	C_17_H_14_N_2_O_3_	14.09	295 (+)	146(24)/177(12)
Cyclopenol	CPL	C_17_H_14_N_2_O_4_	6.68	311 (+)	146(26)/177(14)
Cyclopiazonic acid	CPA	C_20_H_20_N_2_O_3_	37.06	337 (+)	182(18)/196(24)
Griseofulvina	GRI	C_17_H_17_C_l1_O_6_	21.32	353 (+)	165(22)/215(20)
Meleagrine	MEL	C_23_H_23_N_5_O_4_	6.46	434 (+)	403(14)/334(22)
Mycophenolic acid	MPA	C_17_H_20_O_6_	21.2	321 (+)	159(38)/207(22)
Ochratoxin A	OTA	C_20_H_18_ClNO_6_	28.63	404 (+)	239(26)/221(36)
Patulin	PAT	C_7_H_6_O_4_	2.54	153 (-)	109(8)/81(12)
Penicillin G	PenG	C_16_H_18_N_2_O_4_S	12.01	335 (+)	202(24)/217(16)
Penicillin V	PenV	C_16_H_18_N_2_O_5_S	15.29	351 (+)	229(16)/257(17)
Penitrem A	PenA	C_37_H_44_ClNO_6_	42.95	634 (+)	558(18)/616(12)
Roquefortine C	RoqC	C_22_H_23_N_5_O_2_	3.09	390 (+)	193(28)/322(20)
Sulochrin	SUL	C_17_H_16_O_7_	14.02	331 (−)	149(16)/299(14)
Viridicatin	VIR	C_15_H_11_NO_2_	21.48	238 (+)	220(16)/165(30)

**Table 2:** Validation parameters (recovery, LOD, LOQ, ME, and R^2^) calculated for chestnuts.

Analyte	Recovery [%] ± SD ^a^	LOD [ng/g]	LOQ [ng/g]	ME [%]	R^2^
AndA	93.4 ± 7.1	16.3	36.2	34.3	0.9994
ChA	79.3 ± 4.2	9.94	33.1	6.68	0.9996
CIT	85.7 ± 8.7	13.7	45.7	17.8	0.9991
CPN	85.7 ± 7.9	8.60	28.7	158	0.9990
CPL	75.0 ± 3.2	0.12	0.41	172	0.9994
CPA	69.4 ± 6.2	11.1	36.9	43.7	0.9975
MEL	73.2 ± 9.3	11.8	39.2	2.70	0.9995
MPA	87.9 ± 11.2	10.9	36.4	28.5	0.9986
OTA	71.5 ± 3.6	13.8	46.1	7.94	0.9989
PenA	83.6 ± 9.4	8.14	27.1	125	0.9982
RoqC	93.0 ± 13.2	11.1	37.1	79.7	0.9993
VIR	76.3 ± 4.9	13.4	44.6	6.04	0.9986
GRI	73.4 ± 2.0	10.4	34.8	187	0.9996
CVD	76.9 ± 16.1	13.0	43.2	2.27	0.9991
SUL	78.4 ± 4.3	10.4	34.7	137	0.9997
PAT	76.4 ± 6.4	3.04	10.1	21.2	0.9972
PenG	80.5 ± 15.1	21.6	72.1	71.8	0.9998
PenV	79.8 ± 9.6	7.85	26.2	73.1	0.9994
AsA	85.9 ± 5.0	8.86	29.5	151	0.9996

^a^ SD = Standard deviation.

**Table 3:** Validation parameters (recovery, LOD, LOQ, ME and R^2^) calculated for hazelnuts.

Analyte	Recovery [%] ± SD ^a^	LOD [ng/g]	LOQ [ng/g]	ME [%]	R^2^
AndA	100.9 ± 16.3	15.2	50.8	35.5	0.9996
ChA	78.8 ± 8.7	15.8	52.5	12.6	0.9992
CIT	82.4 ± 14.6	14.9	49.7	8.85	0.9995
CPN	87.9 ± 2.4	9.84	32.8	173.2	0.9993
CPL	84.4 ± 13.5	0.09	0.31	186.5	0.9997
CPA	88.2 ± 6.4	31.8	52.9	30.6	0.9938
MEL	82.9 ± 7.1	9.4	31.5	2.38	0.9995
MPA	88.3 ± 5.3	12.7	42.4	8.93	0.9997
OTA	67.9 ± 9.2	9.1	30.4	6.70	0.9997
PenA	92.4 ± 10.3	8.4	28.1	121.3	0.9996
RoqC	64.6 ± 3.1	12.1	40.4	204.0	0.9996
VIR	74.8 ± 7.5	8.7	28.9	5.61	0.9995
GRI	104.2 ± 1.6	8.8	29.3	236.0	0.9997
CVD	88.4 ± 11.3	7.7	25.6	1.72	0.9996
SUL	89.3 ± 2.5	7.1	23.7	98.9	0.9996
PAT	76.6 ± 9.9	10.3	34.5	93.5	0.9999
PenG	85.6 ± 6.1	13.3	44.5	48.9	0.9979
PenV	101.1 ± 4.9	5.6	18.8	66.8	0.9996
AsA	90.0 ± 12.4	14.1	47.1	132.5	0.9975

^a^ SD = Standard deviation.

**Table 4:** Validation parameters (recovery, LOD, LOQ, ME and R^2^) calculated for walnuts.

Analyte	Recovery [%] ± SD ^a^	LOD [ng/g]	LOQ [ng/g]	ME [%]	R^2^
AndA	68.2 ± 5.7	34.4	57.3	10.9	0.9996
ChA	70.2 ± 8.6	9.6	31.9	0.69	0.9997
CIT	87.4 ± 15.7	6.88	22.9	1.79	0.9992
CPN	78.5 ± 3.5	8.44	28.1	43.9	0.9973
CPL	78.4 ± 5.8	0.10	0.34	29.4	0.9997
CPA	98.1 ± 2.7	13.7	45.6	9.73	0.9987
MEL	72.2 ± 6.0	15.3	51.0	0.62	0.9993
MPA	88.1 ± 8.4	10.3	34.2	0.57	0.9995
OTA	80.6 ± 5.0	11.4	38.1	0.69	0.9993
PenA	84.1 ± 4.5	12.4	41.3	40.5	0.9993
RoqC	88.3 ± 2.0	15.0	50.0	50.5	0.9992
VIR	77.1 ± 1.9	6.06	20.19	1.86	0.9989
GRI	78.0 ± 6.8	7.54	25.14	84.0	0.9994
CVD	72.2 ± 8.8	9.05	30.2	0.61	0.9983
SUL	82.3 ± 5.2	15.1	50.2	4.68	0.9981
PenG	97.9 ± 5.6	17.5	58.2	87.6	0.9974
PenV	81.5 ± 3.7	9.88	32.9	97.4	0.9995
AsA	79.8 ± 6.0	13.8	46.1	143	0.9993

^a^ SD = Standard deviation.

**Table 5:** Validation parameters (recovery, LOD, LOQ, ME and R^2^) calculated for almonds.

Analyte	Recovery [%] ± SD ^a^	LOD [ng/g]	LOQ [ng/g]	ME [%]	R^2^
AndA	74.4 ± 11.6	14.1	46.9	12.3	0.9994
ChA	83.8 ± 25.2	11.8	39.4	1.02	0.9992
CIT	84.2 ± 7.4	8.32	27.7	109	0.9990
CPN	73.0 ± 2.2	15.8	52.7	74.6	0.9997
CPL	90.8 ± 8.3	0.14	0.48	86.2	0.9991
CPA	88.2 ± 6.8	10.4	34.6	7.96	0.9997
MEL	85.3 ± 8.8	10.3	34.3	1.00	0.9993
MPA	84.7 ± 9.7	10.6	35.2	0.76	0.9989
OTA	85.2 ± 4.9	3.09	10.3	0.87	0.9994
PenA	66.3 ± 11.7	11.0	36.8	119	0.9996
RoqC	81.9 ± 4.1	11.1	36.9	92.3	0.9995
VIR	76.9 ± 3.6	6.80	22.7	1.34	0.9991
GRI	71.6 ± 1.4	11.6	38.5	14.7	0.9997
CVD	96.2 ± 3.1	10.1	33.5	0.82	0.9997
SUL	79.0 ± 5.2	7.49	25.0	8.43	0.9994
PenG	88.4 ± 10.6	27.8	92.7	77.8	0.9876
PenV	80.7 ± 9.4	4.92	16.4	92.8	0.9987
AsA	91.9 ± 12.9	4.93	16.4	171	0.9993

^a^ SD = Standard deviation.

**Table 6:** Precision of the method validation for the metabolites spiked in the matrices.

Analyte	Intra-Day CV% ^a^					Inter-Day CV%		
	Chestnuts	Hazelnuts	Walnuts	Almonds	Chestnuts	Hazelnuts	Walnuts	Almonds
AndA	12.3	11.1	11.6	13.9	12.3	11.1	11.6	13.9
AsA	11.8	13.3	9.9	10.8	11.8	13.3	9.9	10.8
ChA	10.6	12.1	12.2	11.4	10.6	12.1	12.2	11.4
CVD	8.2	10.5	14.9	14.7	8.2	10.5	14.9	14.7
CIT	14.8	11.5	8.4	6.1	14.8	11.5	8.4	6.1
CPN	1.9	7.0	9.6	11.5	1.9	7.0	9.6	11.5
CPL	7.9	10.3	8.1	8.9	7.9	10.3	8.1	8.9
CPA	12.0	10.8	10.8	13.3	12.0	10.8	10.8	13.3
GRI	7.8	11.6	10.3	9.2	7.8	11.6	10.3	9.2
MEL	8.2	13.6	13.0	14.1	8.2	13.6	13.0	14.1
MPA	4.4	11.0	12.0	11.1	4.4	11.0	12.0	11.1
OTA	6.7	8.0	8.2	13.1	6.7	8.0	8.2	13.1
PAT	11.9	13.0	- ^b^	9.8	11.9	13.0	-	10.5
PenG	12.8	12.4	10.0	13.3	12.8	12.4	10.0	13.3
PenV	13.4	11.0	14.7	10.4	13.4	11.0	14.7	10.4
PenA	5.9	8.7	13.7	4.5	5.9	8.7	13.7	4.5
RoqC	6.8	11.1	8.1	8.9	6.8	11.1	8.1	8.9
SUL	10.6	10.7	10.1	14.0	10.6	10.7	10.1	14.0
VIR	14.9	15.0	14.8	13.4	14.9	15.0	14.8	13.4

a CV% = Coefficient of variability; b Not evaluated.

**Table 7:** Mycotoxin contamination in commercial samples.

Analyte	Chestnuts (n * = 8)			Hazelnuts (n = 13)			Walnuts (n = 9)			Almonds (n = 11)		
	Positive Samples (%)	Min ^a^ (μg/kg)	Max ^b^ (μg/kg)	Positive Samples (%)	Min (μg/kg)	Max (μg/kg)	Positive Samples (%)	Min (μg/kg)	Max (μg/kg)	Positive Samples (%)	Min (μg/kg)	Max (μg/kg)
AndA	62.5	2.8 ± 0.3	237.0 ± 10.2	0	-	-	22.2	37.8 ± 3.5	46.7 ± 2.6	0	-	-
AsA	0	-	-	0	-	-	0	-	-	0	-	-
ChA	62.5	64.5 ± 4.4	3763.5 ± 109.3	15.4	7.6 ± 0.5	29.2 ± 1.9	0	-	-	0	-	-
CIV	0	-	-	0	-	-	0	-	-	0	-	-
CIT	0	-	-	0	-	-	0	-	-	0	-	-
CPN	62.5	2.7 ± 1.1	105.7 ± 19.1	15.4	1.32 ± 0.08	1.37 ± 0.1	44.4	<LOQ	5.72 ± 0.8	18.2	2.16 ± 0.1	2.51 ± 0.1
CPL	62.5	17.6 ± 4.7	1291.0 ± 46.5	76.9	11.02 ± 2.1	21.45 ± 6.4	66.7	10.6 ± 0.5	83.6 ± 3.8	54.5	10.04 ± 0.2	30.17 ± 16.0
CPA	0	-	-	0	-	-	0	-	-	0	-	-
GRI	0	-	-	0	-	-	0	-	-	0	-	-
MEL	0	-	-	0	-	-	0	-	-	0	-	-
MPA	0	-	-	7.7	2.7 ± 0.1	2.7 ± 0.1	11.1	2.6 ± 0.5	2.6 ± 0.5	18.2	2.18 ± 0.0	2.39 ± 0.2
OTA	0	-	-	0	-	-	0	-	-	0	-	-
PAT	0	-	-	0	-	-	0	-	-	0	-	-
PenG	0	-	-	0	-	-	0	-	-	0	-	-
PeV	0	-	-	0	-	-	0	-	-	0	-	-
PenA	25.0	47.4 ± 3.6	67.3 ± 1.1	0	-	-	0	-	-	0	-	-
Roq C	62.5	1.0 ± 0.3	179.8 ± 11.8	7.7	<LOQ	<LOQ	11.1	118.9 ± 33.5	118.9 ± 33.5	9.1	<LOQ	<LOQ
SUL	62.5	13.4 ± 1.1	986.1 ± 10.6	0	-	-	0	-	-	0	-	-
VIR	0	-	-	0	-	-	1.11	151.4 ± 64.3	151.4 ± 64.3	9.1	3.1 ± 0.2	3.1 ± 0.2

* n total number of samples for each nuts, <LOQ below the lower limit of quantification; ^a^ Lowest determined concentration; ^b^ Highest determined concentration.

## Supplementary Materials

The following are available online at https://www.mdpi.com/2072-6651/12/5/307/s1, Table S1. Samples name, city of purchase, sample origin and purchase period about matrices used for chemical analysis in this study, Table S2. Strain name, species, accession numbers (AN) and references for strains used for artificial inoculation of nuts used in this study.
