# Supplementary material for: HPLC-MS/MS Method for the Detection of Selected Toxic Metabolites Produced by Penicillium spp. in Nuts
Source: Toxins (Basel). 2020 May 8;12(5):307. doi: 10.3390/toxins12050307 (PMC7290882; doi:10.3390/toxins12050307)
Supplement: Supplementary file 1 [file toxins-12-00307-s001.pdf]

Supplementary Material: HPLC-MS/MS Method for the Detection of Selected Toxic Metabolites Produced by *Penicillium* spp. in Nuts

Davide Spadaro, Giovanna Roberta Meloni, Ilenia Siciliano, Simona Prencipe and Maria Lodovica Gullino

Table S1. Samples name, city of purchase, sample origin and purchase period about matrices used for chemical analysis in this study.

Nuts	Number samples	City of purchase	Sample origin	Purchase period
**Samples used for the inoculation and validation**				
Chestnuts	10	Turin	Italy	October 2017
Hazelnut	10	Turin	Italy	October–November 2017
Walnuts	10	Turin	Italy	November 2017
Almonds	10	Turin	Italy	November 2017
**Commercial samples**				
Chestnuts	8	Turin	Italy	October 2018
Hazelnuts	13	Turin	Italy, Turkey and USA	between December 2018 and March2019
Walnuts	9	Turin	Italy, France, Moldova, Argentina and Australia.	between December 2018 and March2019
Almonds	11	Turin	Italy, Spain and USA	between December 2018 and March2019

Table S2. Strain name, species, accession numbers (AN) and references for strains used for artificial inoculation of nuts used in this study.

Species	Strain name	ITS (AN)	BenA (AN)	CaM (AN)	Reference (AN)
P. bialowiezense	B1	MG821357	MF100873	MF100893	Prencipe et al. (2018)
P. brevicompactum	Cas18	MG821358	MF100870	MF100890	Prencipe et al. (2018)
P. crustosum	Cas34	MG778547	MG778538	MG778542	This work
P. expansum	POX2	MG778546	MG778536	MG778540	This work
P. glabrum	E3	MG778544	MG778539	MG778543	This work
P. solitum	XF	MG821373	MF100861	MF100881	Prencipe et al. (2018)
